# A Study on the Microbiological Status of Mineral Drinking Water

**DOI:** 10.2174/1874285801711010031

**Published:** 2017-04-28

**Authors:** Faria Y. Aditi, Shafkat S. Rahman, Md. M. Hossain

**Affiliations:** Department of Mathematics and Natural Sciences, BRAC University, Dhaka-1212, Bangladesh

**Keywords:** Mineral water, *Staphylococcus*, Pathogens, heterotrophic plate count (HPC)

## Abstract

**Introduction:**

Water-borne diseases constitute a major health burden in Bangladesh. The objective of this study was to assess the overall quality of mineral water samples that obtained from different shops of Dhaka city.

**Material and Methods:**

To achieve the above-mentioned objective, methods of heterotrophic plate count (HPC) and total coliform count (TCC) were applied. Moreover, isolated colony from mineral water samples were characterized by using biochemical and antimicrobial susceptibility tests.

**Results:**

Different water samples showed different HPC ranged from 1.0×10 to 8.00×10^2^. Antimicrobial sensitivity test of some selected bacteria viz *S. intermedius, S. aureus, S. felis* and *S. Saccharolyticus* were performed. It was observed that *Staphylococcus* spp. isolates were susceptible to erythromycin, tetracycline, norfloxacin and ciprofloxacin. Furthermore, a few *Staphylococcus* spp. isolates were intermediate resistant to penicillin and oxacillin. However, most of the *Staphylococcus* spp. isolates were resistant to cefixime.

**Conclusion:**

The results indicate that mineral water serves as a reservoir of various bacteria and that people in Dhaka city, who are the consumers of these water, might get diseases. This study emphasizes the need for elaborated microbiological examinations of mineral drinking water commonly used in Dhaka city.

## 1 INTRODUCTION

The consumption of mineral water has been substantially increasing all over the world in since decade [1]. The increase also has happened in the countries where tap water is used as drinking water. According to the World Health Report (2002) [2], every year more than 3.4 million individuals die as a result of water related maladies demonstrating these as the leading cause of disease and death around the world. In the disease-prone, humid, tropical region of Bangladesh, episodes of diarrheal diseases, frequently on a pandemic scale, are not uncommon and the conceivable part of water-borne pathogens in these outbreaks has been underlined. Among waterborne infections of bacterial origin typhoid fever, bacillary dysentery and diarrhea are normal in Bangladesh. The pathogenic most frequently transmitted though water, are those which cause infection of the intestinal tract, namely typhoid, paratyphoid diarrhea, dysentery and cholera [3]. Water borne diseases constitute a major health burden in Bangladesh. According to Bangladesh health and injury report on children under 5 in 2005, children die every year from diarrhea [4].

Health effects connected with water supplies in developing nations are assessed to be based on four bacterial markers of tropical drinking-water quality (fecal coliforms, *Escherichia coli*, *Enterococci* and fecal *Streptococci*) and their relationship to the prevalence of diarrheal disease in Cebu, Philippines [5]. The present study was conducted to identify fecal coliforms and pathogenic bacteria e.g. *Escherichia coli*, *Enterobacter*, *Staphylococcus aureus*, *Pseudomonas* and *Vibrio* species (*V. cholerae, V. parahaemolyticus, V. mimicus* and *V. alginolyticus*) from mineral water in Dhaka city. The study endeavored to answer four objectives:

- Bacterial load analysis of mineral water samples collected from different areas of Dhaka city.
- Physiochemical quality analysis of collected samples.
- Cultural and biochemical examinations of isolates.
- Antibiotic test result of isolated organisms.

### 1.1 Pathogens of Mineral Water

#### 1.1.1 Escherichia coli

*E. coli* have been identified on the basis of different virulence factors. Enterotoxigenic *E. coli* (ETEC) produces heat-labile or heat-stable enterotoxin, or both toxins simultaneously, and is an important cause of diarrhea in developing countries, especially in young children. Infection with enteropathogenic *E. coli* (EPEC) has been associated with severe, chronic, non-bloody diarrhea, vomiting and fever in infants. Enteroinvasive *E. coli* (EIEC) causes watery and occasionally bloody diarrhea where strains invade colon cells by a pathogenic mechanism similar to that of *Shigella * [6].

#### 1.1.2 Vibrio Spp.

There are 30 species in the genus *Vibrio*; thirteen of these are pathogenic to humans, including *V. cholerae, V. mimicus, V. fluvialis, V. parahaemolyticus, V. alginolyticus,* and *V. vulnificus*, all of the pathogenic. *Vibrio*s have been reported to cause foodborne and waterborne diseases, although *V. cholera* 01, *Vibrio parahaemolyticus*, and *V. vulnificus* are considered the most significant agents [7].

#### 1.1.3 Staphylococcus Spp.

Members of the *Staphylococcus* frequently colonize the skin and upper respiratory tracts of mammal sand birds. *Staphylococcal* toxins are a common cause of food poisoning by colonizing improperly stored food items. The most common sialadenitis is caused by *Staphylococci*, as bacterial infections [8].

#### 1.1.4 Pseudomonas Spp.

*Pseudomonadaceae* containing 191 validly described species. The members of the genus demonstrate a great deal of metabolic diversity, and consequently are able to colonize a wide range of niches. *P. aeruginosa* flourishes in hospital environments, and is a particular problem in this environment, since it is the second-most common infection in hospitalized patients (nosocomial infections) [9].

## 2 MATERIAL AND METHODS

### 2.1 Place of Study and Sample Collection

Ten commercially available mineral water samples were aseptically collected from many shops of Mohakhali and Cantonment area in Dhaka city in two different seasons, based on the correlation between seasonal influence and water-borne microbial activity [10], from August 2015 to January 2016 (rainy to winter season). In both cases, sample mineral water bottles were labeled in the field and transported to the laboratory and were processed in the Microbiology, Biotechnology and Molecular Biology Laboratory of the Department of Mathematics and Natural Sciences of BRAC University [11] within three hours of collection. The samples were storage in -4°C. Then different physicochemical parameters (pH and conductivity) were measured by using pH meter and conductivity meter and were inoculated into three different and selective agar media to detect the presence of pathogenic bacteria and total count respectively.

### 2.2 Isolation of Bacteria from Sample Water

Three different selective agar media MacConkey, Xylose-Lysine-Deoxycholate or XLD and M-FC agar media [12] were used for isolation of *Escherichia coli*, *Enterobacter*, *Staphylococcus aureus*, *Pseudomonas* and *Vibrio *species [13], the most abundant and severe pathogens in drinking water [14-15], respectively. Nutrient agar media (Peptone 5 g/L, NaCl 5 g/L, Beef extract 3 g/L, Agar 15 g/L) are used for total heterotrophic count of organisms. 0.2 microliter and 10:1 dilutions of the samples were spread on Nutrient agar plate and different selective media. All the plates were then incubated at 37°C for 20 to 36 hours. M-FC plate was incubated in 45°C. After incubation, every plate was observed carefully. Colony morphology of various isolates were examined and recorded on the basis of size, form, pigmentation, margin, elevation and opacity for evaluation of microscopic character, pure colony of each isolates was picked and Gram staining was performed. The size, shape, arrangement and Gram reaction properties of isolates were carefully observed.

### 2.3 Biochemical Characteristics of the Isolates

#### 2.3.1 Catalase Test

A microscopic slide was placed inside a petri dish. Using a sterile inoculating loop, a small amount of microorganism from 24-hour pure culture was placed onto the microscopic slide. 3% H_2_O_2_ solution was added to each of the slides and a portion of the bacterial colony was mixed with it. Production of bubble indicated the presence of catalase enzyme in the bacteria [16].

#### 2.3.2 Oxidase Test

A portion of the colony was picked up with a tooth pick and rubbed on a strip of a filter paper impregnated with oxidase reagent (1% aqueous solution of N’N’N’N’12 tetramethyl-p-phenylenediaminedihydrochloride). Oxidase test indicates positive by the presence of dark purple color within 10 seconds [17].

#### 2.3.3 Triple Sugar Iron (TSI) Agar Test

Triple sugar iron test was done to differentiate among the different groups or genera of the *Enterobacteriaceae *based on the ability to reduce sulfur and ferment carbohydrates. Slants were prepared in the test tubes by autoclaving. Using sterile technique; small amount of the experimental bacteria from 24-hours old pure culture was inoculated into the tubes by means of a stab and streak inoculation method with an inoculating needle. The screw caps were not fully tightened and the tubes were incubated for 24 hours at 37°C. Fermentation is indicated by yellowing of the butt and the slant of Triple Sugar Iron (TSI) agar media. If gas was formed during the fermentation, it is shown in the butt either by the formation of bubbles or cracking of the agar [18, 19].

#### 2.3.4 Motility Indole Urea (MIU) Test

Following incubation for 18-24 hrs at 37°C, the colony in tube was observed for the presence of motile organisms. Production of cherry red reagent layer after introduction of Kovac’s reagent in MIU medium indicates Indole positive reaction [19-20].

#### 2.3.5 Citrate Utilization Test

Citrate utilization test was done to differentiate among enteric organisms on the basis of their ability to ferment citrate as a sole source of carbon by the enzyme citrate permease. Simmons citrate agar slants of 2 ml in each vial was prepared by autoclaving at 15 psi 121°C. Using sterile technique, small amount of the experimental bacteria from 24-hours old pure culture was inoculated into the vials by means of a streak inoculation method with an inoculating needle and the vials were incubated for 48 hours at 37°C. Following incubation, citrate positive culture was recognized by the presence of growth on the surface of the slant of Simmons citrate agar and deep Prussian blue coloration of the medium [18-19].

#### 2.3.6 Methyl Red (MR) Test

Methyl red test was done to determine the ability of the bacteria to oxidize glucose with the production and stabilization of high concentration of acid end products. Using sterile technique, small amount of the experimental bacteria from 24-hours old pure culture was inoculated into the tubes contained MR-VP broth (7 ml) by means of a loop inoculation method with an inoculating loop and the tubes were incubated for 24 hours at 37°C. After 24 hours 3.5 ml from the culture tubes were transferred to clean test tubes for Voges-Proskauer test and the remaining broth were re-incubated for additional 24 hours. After 48-hour incubation 5 drops of methyl red indicator was added directly into the remaining aliquot of the culture tubes to carefully observe the immediate development of a red color. [18-19].

#### 2.3.7 Voges-Proskaur (VP) Test

Voges Proskauer test was done to differentiate further among enteric organisms such as *E. coli, E. aerogenes*, and *K. pneumoniae *by determining the capability of the organisms to produce non-acidic or neutral end products such as acetylmethylcarbinol. To the aliquot of MR-VP broth after 24-hour incubation, 0.6 ml (12 drops) of 5% alpha naphthol (Barritt’s reagent A) was added followed by 0.2 ml (4 drops) of 40% KOH (reagent B). The tube was gently shaken to expose the medium to atmospheric oxygen (30 seconds-1 minute) and the medium was allowed to remain undisturbed for 10-15 minutes. The test was read, but not beyond, one hour following the addition of the reagents. [19, 21].

#### 2.3.8 Nitrate Reduction Test

Aseptically inoculate nitrate stock with a heavy growth of test organism. Following 24 to 48-hours incubation, 5 drops of reagent A and 5 drops of reagent B added to each broth. Nitrate Reduction can be Positive: (Red after sulfanilic acid + alpha-naphthylamine; no color after zinc) or Negative: (No color after sulfanilic acid + alpha-naphthylamine followed by Red after zinc) [19].

#### 2.3.9 Gelatin Hydrolysis Test

Gelatin hydrolysis test was done to detect the ability of the bacteria to produce gelatinase. It separates the gelatinase positive, pathogenic *Staphylococcus aureus* from the gelatinase negative, nonpathogenic *Staphylococcus epidermidis*. All the ingredients of the nutrient gelatin medium were mixed and gently heated to dissolve. Three milliliter from the media was dispensed in glass vials and autoclaved. The tubed medium was allowed to cool in an upright position before use. Using sterile technique, a heavy inoculum of 24-hour old culture bacteria was stab inoculated into the tubes with an inoculating needle. The glass vials were then incubated at 37°C and observed up to for 1 week [22].

#### 2.3.10 Blood Agar or Coagulase Test

By giving a culture medium enriched with red blood cells, it is possible to figure out whether a bacterium can demolish the cells and whether it can process the hemoglobin inside. A nutrient medium augmented with the addition of 5% (vol/vol) sterile defibrinated sheep blood. For this test 24-hours incubation at 37°C is considered sufficient. Medium surrounding colonies in the plate was observed. If the culture showed a darkening or discoloration of the medium in the vicinity of growth demonstrates then it is α-hemolysis. After incubation, the plates were observed for clear halos around colonies and under growth to prove gamma, beta and alpha hemolysis [23].

#### 2.3.11 Casein Hydrolysis Test (Plate)

After inoculated the organism on the sterilized plate either a straight line or a zigzag it was incubated at 25° or 37°C for 24 hours [24]. For result, the plate lifted to the light to see the zones. If the result is Positive reactions may be recorded as strong or weak +ve reactions. A zone of clearing around the growth area identifies the presence of caseinase.

#### 2.3.12 Starch Hydrolysis

The purpose Starch hydrolysis test was to check if the microorganism can utilize starch, as a source of carbon and energy for growth. Utilization of starch is accomplished by an enzyme called α-amylase. The inoculated sterile plate of starch agar is incubated at 35-37°C for 48 hours. Iodine reagent is then added to surge the growth. Iodine reagent is added after incubation to flood the surface of the plate. The dropper was set above the plate and adds the reagent to the culture. Changes in the plate was monitored. The starch in the plate was changed to blue-brown by the iodine reagent. Zones where starch has been processed by bacterial growth display clear halos in the midst of the dark plate, demonstrating a positive α-amylase, or starch hydrolysis test. Plates were containing bacteria without α-amylase was uniformly dark [18, 25].

#### 2.3.13 Mannitol Salt Agar

The media is selective and differential function for *Staphylococcus aureus*. Gram positive *cocci*, particularly *Staphylococci *ferment mannitol and exhibit a yellow zone by color change the pH indicator phenol red, surrounding their growth. Non-mannitol fermenters give colorless zone [26].

### 2.4 Antimicrobial Susceptibility

Susceptibility and resistance of different antibiotics was measured in vitro by employing the Kirby-Bauer method [27]. A suspension of test organism was prepared in nutrient broth by overnight culture for 24 hours at 37ºC and 7.0 pH. The broth was streaked using by sterile glass spreader homogenously on the medium [19]. Antibiotic disc was applied aseptically to the surface of the inoculated plates at an appropriate special arrangement with the help of a sterile forceps on Mueller-Hinton agar plates. The plates were then inverted and incubated at 37ºC for 24 hours. The diffusion discs with antimicrobial drugs were placed on the plates and incubated for 24 hours at 37ºC. 18 antibiotics discs were used. Sterile glass spreader was used to spread the culture homogenously on the medium. Antibiotic disc was applied aseptically to the surface of the inoculated plates at an appropriate special arrangement with the help of a sterile forceps. The plates were then inverted and incubated at 37°C for 24 hours. After incubation, the plates were examined and the diameters of the zone of complete inhibition were observed.

All the biochemical and antibiotic tests were aseptically performed using two containers (plate or tube) of same sample, with repeated experiments. The results were the average of collected data.

### 2.5 Qualitative Assessment of Bottled Water

To acquire a public perception regarding bottled water in the Dhaka city of Bangladesh, a small questionnaire survey, with about 100 participants, was designed (questionnaire not shown) [28]. The survey conducted in the Mohakhali area (in campus and vicinity of BRAC University).

### 2.6 Maintenance and Preservation of Isolates

Typical and atypical colonies of bacterial isolates were picked up and streaked on nutrient agar plate. After 24 hours of incubation at 35°C, all the isolates were inoculated individuals containing nutrient agar slant with sterile paraffin and preserved at -4°C.

## 3 RESULTS

**Fig. (1):**
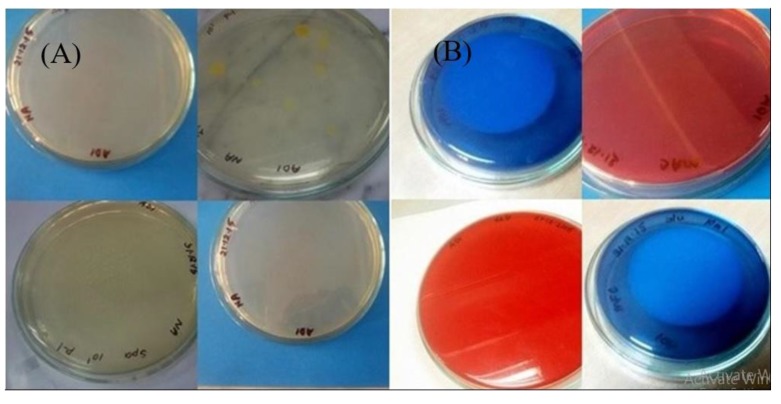
(A) Bacterial colonies on in nutrient Agar (NA) media, (B) No bacterial colonies were grown on the selective media, Membrane Faecal-coliform, Xylose Lysine Deoxycholate agar and MacConkey agar media.

**Fig. (2):**
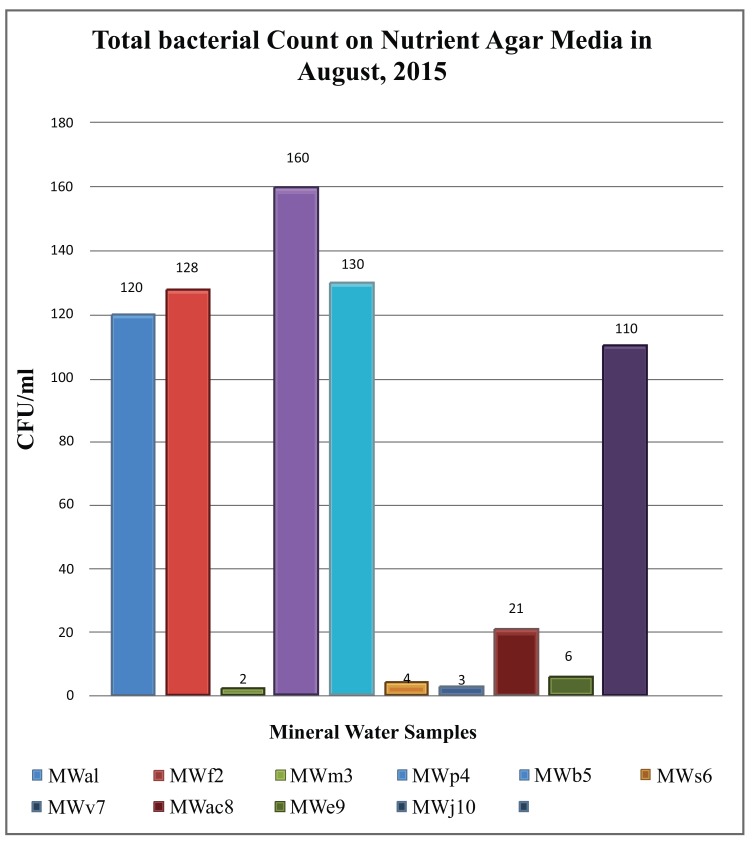
Total bacterial count on nutrient agar media in August 2015.

**Fig. (3):**
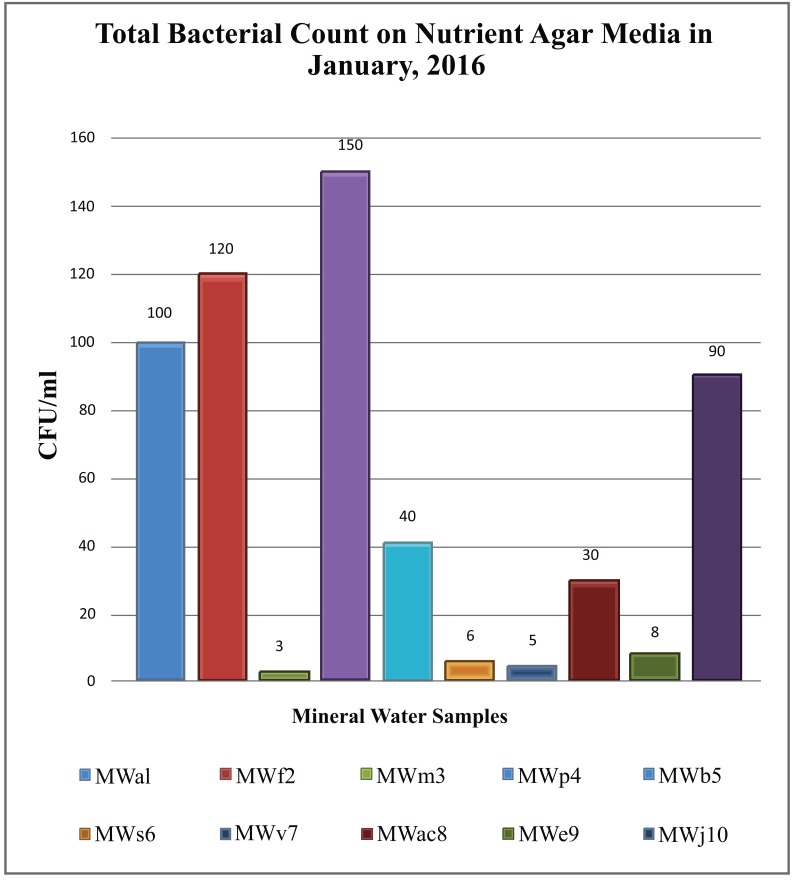
Total bacterial count on nutrient agar media in January 2016.

While comparing the overall result, it was found that the bacterial count only found on nutrient agar media. Nutrient agar plates were used for the calculations of total viable count (TVC). Bacterial colonies of different morphology and color were observed (Fig. **1A**). There was no growth on MacConkey agar, M-FC agar and XLD agar media. Heterotrophic plate count (HPC) and total coliform count (TCC) are frequently used to evaluate the general microbiological quality of mineral water. But no TCC was observed in the present study (Fig. **1B**). The HPC of bottled water of ten different brands conducted in this study. HPC was found the lowest (1.0×10) in MWm3 bottled mineral water but the highest (8.0×10^2^) in MWp4 in this study (Table **1**). Maximum count was observed in the sample of mineral water during rainy season (Fig. **2**). The HPC of mineral water of ten different brands were re-conducted. HPC was found the lowest (1.5×10) in MWm3 bottled water but the highest (7.50 ×10^2^) in MWp4 bottled water once again (Table **1**). Maximum count was observed in the sample of mineral water during winter season (Fig. **3**). The maximum pH was detected 7.4 in the samples, while the minimum pH was seen 6.7 (Table **1**). In this study, MWm3 bottled water was observed to be best in terms of microbiological quality when compared with other brands of mineral water available in Dhaka city of Bangladesh.

The isolates of colony were named according to respective samples’ number and name (Table **1**). After that these isolates colony was used for biochemical tests to find organism and also antibiotic test for sensitivity.

**Fig. (4):**
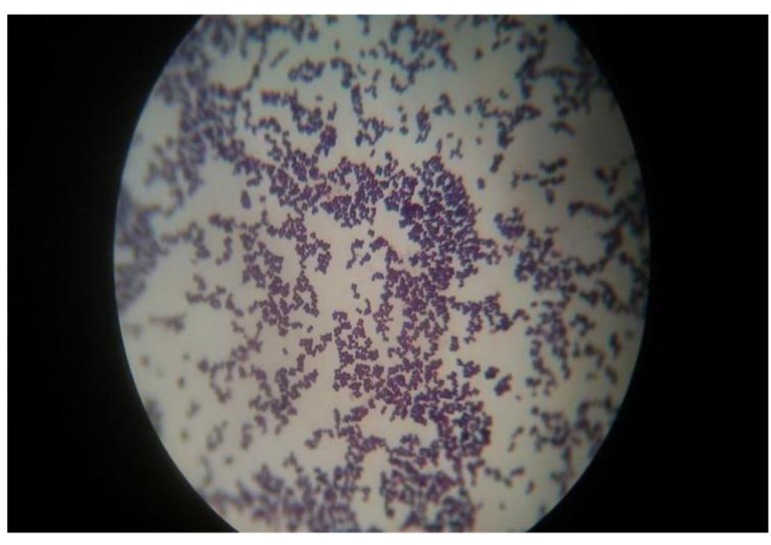
Unknown organisms were showing gram positive and purple color by Gram staining method (100X).

Gram staining revealed that all the isolates organisms were *Staphylococcus* spp. (Fig. **4**). For this biochemical test was observed to find out organism’s name.

### 3.1 Biochemical Characteristics of the Isolates

The colony characteristics of the isolated colony were differing from each other. All the biochemical tests result of twelve isolated organisms were observed and the organisms were identified as different genus of *Staphylococcus* spp. (Table **2**).

Catalase test Fig. (**5**), MR test showed all-positive reactions. Oxidase test Fig. (**5**), TSI agar test, Blood agar test Fig. (**6**) and Starch hydrolysis test showed all-negative results. Citrate utilization test, VP test, Gelatin hydrolysis test and Mannitol salt agar test produced negative results by most isolates. Mixed results observed for MIU test, Nitrate reduction test and Casein hydrolysis test (Table **2**).

Aggregating all biochemical test results and database check by abis online software [29], out of twenty samples in two different seasons of different areas mineral water were found to contain *S. intermedius, S. aureus, S. felis, S. auricularis, S. hominis* and *S. saccharolyticus* (Table **2**). Out of 12 isolates three different isolates confirmed the growth of *S. intermedius* and *S. saccharolyticus,* two showed the growth of *S*. *auricularis* and *S. felis.* Another two isolates confirmed as *S. aureus and S. hominis.* Important biochemical test results are delineated in the Figs. (**3**, **4**) and in (Table **2**).

### 3.2 Antimicrobial Susceptibility

Of the 12 isolates, antibiotic resistance pattern of 7 isolates were investigated (Table **4**). *S. intermedius, S. aureus, S. felis* and *S. saccharolyticus* were taken for antibiotic test. *Staphylococcus aureus* was able to acquire resistance easily; therefore, it is a good bio-indicator model for surveillance studies of antimicrobial resistance. Antimicrobial resistance testing was performed by disc diffusion method using 18 different antibiotics. In antimicrobial susceptibility test, most of the organisms were intermediate resistant to penicillin, oxacillin, clindamycin and susceptible resistant to erythromycin, vancomycin, trimethoprim-sulfamethoxazole, gentamicin, tetracycline, norfloxacin chloramphenicol, moxifloxacin, nitrofurantoin, ciprofloxacin, rifampin, minocycline, levofloxacin and resistance to cefixime, nitrofurantoin (Tables **3**, **4** and Fig. (**7**)).

### 3.3 Qualitative Assessment of Bottled Water

In this study, the age group belonged to 12-24 years favored bottled water mostly (60%). However, 66.7% undergraduate student favored bottled water for their everyday consumption. The cause of preference of bottled water was health awareness, which is corroborated by 70% of the total peoples. The criteria to be good bottled water were taste (43.3%). The bottled water quality was satisfactory to 40% of the peoples in this study on the basis of people’s satisfaction, perception and expenditure on bottled water quality. On the other hand, the percentage of dumping of bottled water after consumption refuse was 33.3%. In addition, monthly expenditure on bottled water was less than taka 300 in 80% of the total people.

## 4 DISCUSSION

The World Health Organization has assessed that up to 80% of all sickness and disease on the planet is created by inadequate sanitation, polluted water or unavailability of water and at least 5 million deaths per year can be credited to water-borne ailments [31].

Bottled water generally receives no further treatment by the consumer before consumption, so its microbiological safety and quality are of paramount importance. The microbiological quality and safety of bottled water is influenced by the microbiological status of the source water and the level of hygiene in the extraction and bottling process.

After performing all required test (microbial culture and biochemical), the results of the study were revealed. A total of 12 isolates were assumed as different species of *Staphylococcus* spp. on the basis of cultural and biochemical characteristics from mineral water samples used in this study (Table **2**).

Microbial count on nutrient agar ranged between 1.0×10 cfu/ml and 8.00×10^2^ cfu/ml. in the rainy season of August 2015 (Table **1**). And January 2016 data showed microbial count ranged between 1.5×10 cfu/ml and 7.50×10^2^ cfu/ml. on the same medium (Table **1**). According to the world health report (2002), drinking water quality specifications world-wide recommend HPC limits 50 cfu/ml in mineral water [2].

The pH ranged between maximum 7.4 to minimum 6.7 in the samples (Table **1**). United State Public Health (USPH) standardized drinking water pH to 7.0 [32].

The fermentation reaction by the isolates of *S. aureus* in basic sugars (lactose and mannitol) were positive. Moreover, MR reaction and catalase tests were also positive for *Staphylococcus aureus*. All the biochemical tests result of twelve isolated organisms were observed and the entire organisms were identified as different genus of *Staphylococcus* spp. (abis online software). Among them seven organisms were taken from different isolated samples for antibiotic test.

The result of sugar fermentation tests corresponds to the previous findings [33-34]. These respective authors reported that albeit *S. aureus* ferments sugars, variation of the results might be due to genetic factors and nature of inhabitant of the organisms. Malaney and Weiser (1962) isolated *S. aureus* from water [35]. Dragas and Tratnik (1975) [36] stated that 21.5% of mineral were contained *S. aureus. *Lin *et al. *(1974) [37] and Mieres and Bastardo (1975) [38] isolated *S. aureus *from mineral water and found that the organism was present in majority of the improved water sources. Likewise, in the present study *Staphylococcus aureus *was detected and found absent in bottled water. The findings of the present study obviously demonstrated that protection of commercial mineral water sources is very important and the avoidance of contamination can promote hygienic quality of mineral water supplies.

In respect to antimicrobial susceptibility testing Kirby-Bauer method allowed for the rapid determination of the efficacy of a drug by measuring the diameter of the zone of inhibition that resulted from diffusion of the agent into the medium surrounding the disc. Most of the *Staphylococcus* spp. isolates were susceptible to erythromycin, tetracycline, norfloxacin and ciprofloxacin. Furthermore, a few *Staphylococcus* spp. isolates were intermediate recalcitrant to penicillin and oxacillin. However, most of the *Staphylococcus* spp. isolates were resistant to cefixime. These findings are in partial agreement with Islam *et al.*, (2010) [39] and Nazir *et al.*, (2005) [40]. Such high rate of multidrug resistant may be because of indiscriminate use of antibiotics, which may eventually have superseded the drug resistant microorganisms from antibiotic saturated environment. In Bangladesh, for a long-time antibiotic is randomly used for treatment purposes. People are not aware about the schedule use of antibiotics. Thus, resistant strains might be emerged by genetic recombination against one or more antimicrobial agent(s).

Qualitative assessment of bottled water of this research work indicated that a decent number of individuals favored bottled water rather than tap water. The discoveries of the study are pretty much like to the previous studies [28, 41].

The findings are in harmony with recent worldwide studies [42-45]. Previous study already scrutinized the Total Dissolve Solids (TDS) level of these commercial mineral waters [46]. Further research is needed to be carried out with increased number of samples for better results and at different location of Dhaka city along with the consideration of total suspended solid (TSS), turbidity, and temperature variation.

## CONCLUSION

Therefore, from the findings of the present study, it may be concluded that a number of people preferred bottled water rather than tap water for their daily consumption. Almost all of the commercial brand bottled mineral water of Bangladesh need to bolster their quality control procedures. MWm3 bottled water found to be superior in terms of microbiological quality to other brands available in Dhaka city. However, it does not confirm that the overall quality of this sample is good because it may contain toxic preservative. Only HPC were found in commercially available mineral water. The isolates of *Staphylococcus* spp. were isolated and characterized from samples using various cultural, morphological investigation, biochemical experiments. However, the following tasks may be scheduled for further study-Molecular characterization of *Staphylocoocus* spp. isolated from bottled mineral water by using PCR, PCE-RFLP, sequencing and so on Genome analysis to have an idea about the genes responsible for pathogenicity and multidrug resistant of *Staphylocoocus* spp. isolated from commercial mineral water.

## Figures and Tables

**Fig. (5):**
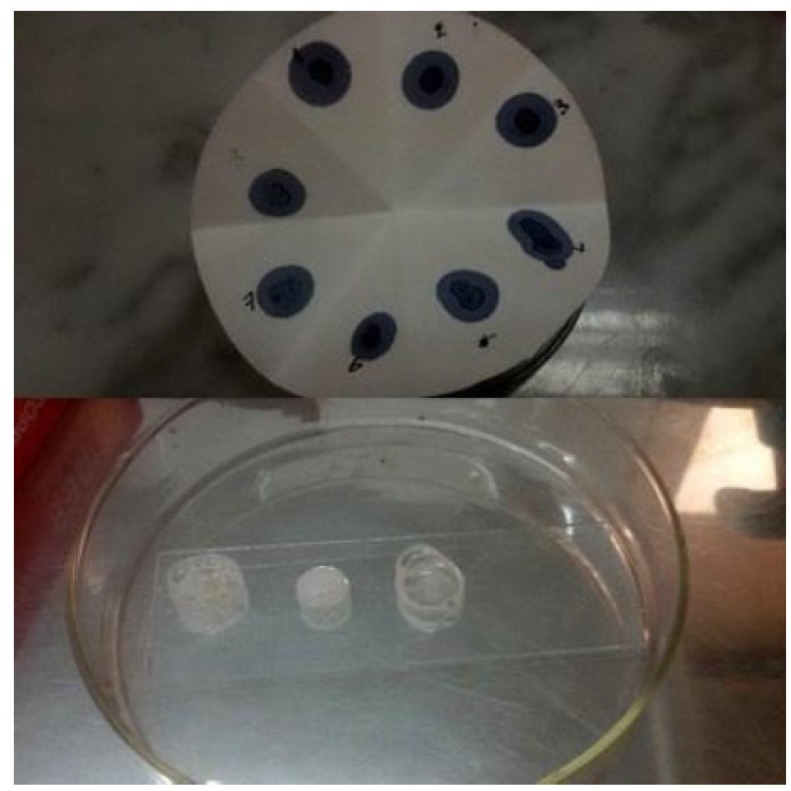
Oxidase and catalase test. Oxidase negative (top) and catalase positive (bottom).

**Fig. (6):**
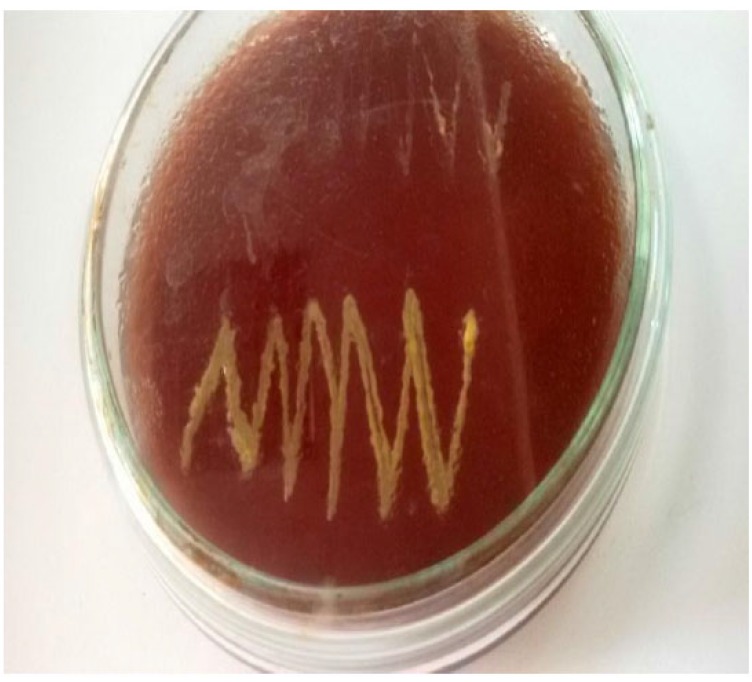
Blood agar test (no hemolysis found).

**Fig. (7):**
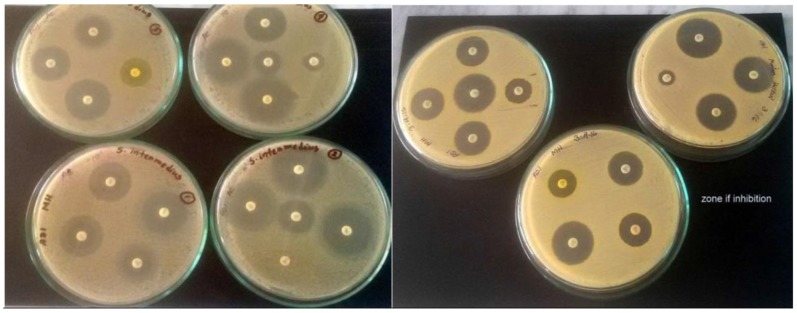
Antimicrobial susceptibility testing of *Staphylococcus aureus, S. felis*, *S. intermedius and S. saccharolyticus* isolated by disc diffusion method.

**Table 1:** pH value of sample mineral waters and microbial load in different media.

**Sample No.**	**Sample** **Name**	**pH**	**Growth on Nutrient Agar** **(CFU/ml)**		**Growth on Mac-Conkey Agar** **(CFU/ml)**		**Growth on Membrane** **Faecal Coliform Agar** **(CFU/ml)**		**Growth on Xylose Lysine Deoxycholate Agar (CFU/ml)**	
			**August 2015**	**January 2016**	**August 2015**	**January 2016**	**August 2015**	**January 2016**	**August 2015**	**January 2016**
1	MWa1	6.9	6.00 ×10^2^	5.00 ×10^2^	NIL	NIL	NIL	NIL	NIL	NIL
2	MWf2	6.7	6.40 ×10^2^	6.00 ×10^2^	NIL	NIL	NIL	NIL	NIL	NIL
3	MWm3	7.4	1.0 ×10	1.5 ×10	NIL	NIL	NIL	NIL	NIL	NIL
4	MWp4	7.4	8.00 ×10^2^	7.50 ×10^2^	NIL	NIL	NIL	NIL	NIL	NIL
5	MWb5	7.1	1.50 ×10^2^	2.00 ×10^2^	NIL	NIL	NIL	NIL	NIL	NIL
6	MWs6	6.8	2.0 ×10	3.0 ×10	NIL	NIL	NIL	NIL	NIL	NIL
7	MWv7	6.8	1.5 ×10	2.5 ×10	NIL	NIL	NIL	NIL	NIL	NIL
8	MWac8	7.0	1.05 ×10^2^	1.5 0×10^2^	NIL	NIL	NIL	NIL	NIL	NIL
9	MWe9	6.9	3.0 ×10	4.0 ×10	NIL	NIL	NIL	NIL	NIL	NIL
10	MWj10	6.9	5.50 ×10^2^	4.50 ×10^2^	NIL	NIL	NIL	NIL	NIL	NIL

**Table 2:** Results of biochemical tests of the isolates collected from nutrient agar.

**Isolate no**	**Sample Name**	**Isolate** **Name**	**Gram Stain**		**TSI**				**MIU**			**Catalase**	**Oxidase**	**Citrate**	**Lactose**	**MR**	**VP**	**Nitrate** **Reduction**	**Gelatin** **Hydrolysis**	**Blood Agar**	**Casein Hydrolysis**	**Starch** **Hydrolysis**	**Mannitol**	**Presumptive** **Organism**
			**+/-**	**Shape**	**BUTT**	**SLANT**	**H_2_S**	**Gas**	**Motility**	**Indole**	**Urease**													
**1.**	**MWa1**	**Mwa1**	**-**	cocci	K	K	**-**	**-**	**+**	**+**	**+**	**+**	**-**	**-**	**-**	**+**	**-**	**+**	**-**	**-**	**+**	**-**	**-**	***S. intermedius***
**2.**	**MWf2**	**MWf2**	**+**	cocci	A	A	**-**	**-**	+	+	+	**+**	**-**	-	+	-	-	-	-	-	-	-	-	***S. auricularis***
**3.**	**MWp4**	**MWp^r^4**	**+**	cocci	A	A	**-**	**-**	-	-	-	**+**	**-**	-	+	+	-	-	-	-	-	-	-	***S. aureus***
**4.**	**MWp4**	**MWp^1^4**	**+**	cocci	K	K	**-**	**-**	-	+	+	**+**	**-**	-	-	+	-	+	-	-	-	-	-	***S. auricularis***
**5.**	**MWb5**	**MWb5**	**+**	cocci	A	A	**-**	**-**	-	+	+	**+**	**-**	-	+	+	-	+	-	-	-	-	-	***S. hominis***
**6.**	**MWs6**	**MWs6**	**+**	cocci	A	A	**-**	**-**	-	-	-	**+**	**-**	-	+	+	-	+	-	-	-	-	-	***S. felis***
**7.**	**MWv7**	**MWv7**	**+**	cocci	A	A	**-**	**-**	+	+	-	**+**	**-**	+	+	+	+	+	+	-	-	-	+	***S. saccharolyticus***
**8.**	**Mwac8**	**MWac^r^8**	**+**	cocci	A	A	**-**	**-**	-	+	+	**+**	**-**	-	+	+	-	+	-	-	+	-	-	***S. saccharolyticus***
**9.**	**Mwac8**	**MWac^1^8**	**+**	cocci	K	K	**-**	**-**	+	+	-	**+**	**-**	-	-	+	-	+	-	-	-	-	-	***S. intermedius***
**11.**	**Mwe9**	**MWe^r^9**	**+**	cocci	K	K	**-**	**-**	+	-	-	**+**	**-**	-	-	-	-	+	-	-	+	-	-	***S. saccharolyticus***
**12.**	**Mwe9**	**MWe^1^9**	**+**	cocci	A	A	**-**	**-**	-	+	+	**+**	**-**	-	+	+	-	+	-	-	-	-	-	***S. felis***
**10.**	**MWj10**	**MWj10**	**+**	cocci	K	K	**-**	**-**	+	-	-	**+**	**-**	-	-	+	-	+	-	-	-	-	-	***S. intermedius***
K = Alkaline reaction, A = Acidic reaction, + = Positive reaction; - = Negative reaction																								

**Table 3:** List of antibiotics and diameter of zone inhibition standard [30].

**Antibiotic Name**	**Disc** **Concentration (**μg**)**	**Diameter of Zone of Inhibition**		
		**Resistant** **<or = nm**	**Intermediate** **nm**	**Susceptible** **= or>nm**
1. Erythromycin	15	≤13	14-22	≥23
2. Penicillin	10	≤19	20-27	≥28
3. Vancomycin	30	≤14	15-16	≥17
4. Trimethoprim- sulfamethoxazole	25	≤10	11-15	≥16
5. Gentamicin	10	≤12	13-14	≥15
6. Oxacillin	1	≤10	11-12	≥13
7. Tetracycline	30	≤14	15-18	≥19
8. Chloramphenicol	30	≤12	13-17	≥18
9. Moxifloxacin	5	≤20	21-23	≥24
10. Norfloxacin	10	≤12	13-16	≥17
11. Nitrofurantoin	30	≤14	15-16	≥17
12. Ciprofloxacin	5	≤15	16-20	≥21
13. Rifampin	5	≤16	17-19	≥20
14. Doxycycline	30	≤12	13-15	≥16
15. Cefixime	5	≤15	16-18	≥19
16. Minocycline	30	≤14	15-18	≥19
17. Levofloxacin	5	≤15	16-18	≥19
18. Clindamycin	2	≤14	15-20	≥21

**Table 4:** Antimicrobial sensitivity pattern of different organism isolated from mineral water.

**Antibiotic Name**	**Isolated sample name**													
	**MWa1**		**MWpr4**		**MWs6**		**MWac18**		**MWj10**		**MWer9**		**MWe19**	
	*S. intermedius*		*S. aureus*		*S. felis*		*S. intermedius*		*S. intermedius*		*S. saccharolyticus*		*S. felis*	
	Zone of Diameter (mm)	Interpretation	Zone of Diameter(mm)	interpretation	Zone of Diameter(mm)	interpretation	Zone of Diameter(mm)	interpretation	Zone of Diameter(mm)	interpretation	Zone of Diameter(mm)	interpretation	Zone of Diameter(mm)	interpretation
**1. Erythromycin**	42	S	32	S	38	S	28	S	31	S	15	I	18	I
**2. Penicillin**	38	S	25	I	40	S	29	S	41	S	45	S	26	I
**3. Vancomycin**	31	S	21	S	31	S	30	S	30	S	32	S	24	S
**4. Trimethoprim-** **sulfamethoxazole**	26	S	25	S	25	S	20	S	35	S	30	S	0	R
**5. Gentamicin**	38	S	26	S	33	S	22	S	31	S	30	S	31	S
**6. Oxacillin**	18	S	11	I	0	R	0	R	0	R	0	R	26	S
**7. Tetracycline**	40	S	29	S	40	S	30	S	41	S	40	S	14	I
**8. Chloramphenicol**	43	S	29	S	44	S	32	S	42	S	42	S	35	S
**9. Moxifloxacin**	40	S	35	S	40	S	35	S	40	S	38	S	42	S
**10. Norfloxacin**	26	S	25	S	23	S	27	S	20	S	25	S	30	S
**11. Nitrofurantoin**	14	R	20	S	14	R	20	S	10	R	10	R	20	S
**12. Ciprofloxacin**	35	S	29	S	31	S	32	S	29	S	30	S	34	S
**13. Rifampin**	45	S	22	S	20	S	24	S	43	S	45	S	20	S
**14. Doxycycline**	40	S	29	S	38	S	30	S	40	S	40	S	20	S
**15. Cefixime**	20	S	0	R	0	R	15	R	0	R	0	R	0	R
**16. Minocycline**	40	S	30	S	41	S	35	S	42	S	43	S	24	S
**17. Levofloxacin**	32	S	28	S	30	S	30	S	28	S	30	S	35	S
**18. Clindamycin**	40	S	20	I	30	S	18	I	38	S	25	S	19	I
*S= Susceptible *R= Resistant *I= Intermediate														

## AVAILABILITY OF DATA AND MATERIALS

Please contact author for data requests.

## LIST OF ABBREVIATIONS

pH: = Negative logarithm of hydrogen ion concentration
spp.: = Species
Conc.: = Concentration
°C: = Degree centigrade
*et al.*: = Associates
*E. coli*: = *Escherichia coli*
ETEC: = Enterotoxigenic *E. Coli*
EPEC: = Enteropathogenic *E. Coli*
EIEC: = Enteroinvasive *E. Coli*
ml: = Milliliter
MR: = Methyl Red
VP: = Voges-Proskauer
HPC: = Heterotrophic Plate Count
TCC: = Total Coliform Count
TVC: = Total Viable Count
